# P-1620. Potential Impact of the Next-Generation COVID-19 mRNA-1283 Vaccine on Post-acute Outcomes of COVID-19

**DOI:** 10.1093/ofid/ofaf695.1797

**Published:** 2026-01-11

**Authors:** Keya Joshi, Michele Kohli, Michael Maschio, Amy Lee, Nicolas Van de Velde, Ekkehard Beck

**Affiliations:** Moderna, Inc, Cambridge, Massachusetts; Quadrant Health Economics Inc, Cambridge, Ontario, Canada; Quadrant Health Economics Inc, Cambridge, Ontario, Canada; Quadrant Health Economics, Cambridge, Ontario, Canada; Moderna, Inc., Cambridge, Massachusetts; Moderna, Inc, Cambridge, Massachusetts

## Abstract

**Background:**

The disease burden associated with COVID-19 continues to significantly impact adults in the United States (US), particularly those aged ≥65 years. While policy discussions often center on the acute phase of severe COVID-19, post-acute outcomes—such as hospital readmissions, post-discharge mortality, and long COVID—likely contribute substantially to the overall burden. This study aimed to estimate the potential impact of hypothetical Fall 2024/25 single-dose vaccination with Moderna's next-generation mRNA-1283 vaccine on post-acute disease burden in US persons aged ≥12 years, compared with currently available mRNA vaccines.

**Methods:**

A dynamic transmission model was adapted to predict COVID-19 incidence, hospitalizations, and post-acute outcomes for the 2024/25 season. The model was calibrated using US data, including age-specific COVID-19 hospitalization and vaccination coverage rates from the 2023/24 season. Post-acute outcomes evaluated included hospital readmissions, post-discharge mortality, and cases of long COVID. Relative vaccine effectiveness of mRNA-1283 compared with current mRNA COVID-19 vaccines mRNA-1273 and BNT162b2 (against infection and hospitalization) was derived from a pivotal Phase 3 trial and an indirect treatment comparison.

**Results:**

Compared to mRNA-1273 and BNT162b2, mRNA-1283 was estimated to prevent in the overall US population an additional 3,132 (vs. mRNA-1273) and 4,517 (vs. BNT162b2) hospital readmissions; 2,604 and 3,757 post-discharge deaths; and 18,450 and 32,382 cases of long COVID, respectively. Approximately 78-79% of readmissions and post-discharge deaths, and 41-42% of long COVID cases were averted among older adults ≥65 years.

**Conclusion:**

These findings suggest that hypothetical Fall 2024/25 mRNA-1283 vaccination could substantially reduce post-acute COVID-19 outcomes, particularly among adults aged ≥65 years. By more effectively preventing acute disease, mRNA-1283 may significantly lower post-acute COVID-19 outcomes compared to current mRNA vaccines. The results underscore the importance of considering post-acute disease burden in vaccination policy and support the potential value of next-generation COVID-19 vaccines like mRNA-1283 in optimizing comprehensive COVID-19 prevention in the US.

**Disclosures:**

Keya Joshi, PhD, Moderna, Inc.: Employee|Moderna, Inc.: Stocks/Bonds (Public Company) Michele Kohli, PhD, Moderna, Inc: Advisor/Consultant|Quadrant Health Economics Inc: Ownership Interest|Quadrant Health Economics Inc: Stocks/Bonds (Private Company) Michael Maschio, MSc, Moderna, Inc: Advisor/Consultant Amy Lee, MSc, PhD, Moderna, Inc: Advisor/Consultant Nicolas Van de Velde, PhD, Moderna, Inc: Stocks/Bonds (Public Company) Ekkehard Beck, PhD, Moderna, Inc: Employee of Moderna, Inc|Moderna, Inc: Stocks/Bonds (Public Company)

